# Supplementary material for: Cardiac Gq Receptors and Calcineurin Activation Are Not Required for the Hypertrophic Response to Mechanical Left Ventricular Pressure Overload
Source: Front Cell Dev Biol. 2021 Feb 15;9:639509. doi: 10.3389/fcell.2021.639509 (PMC7917224; doi:10.3389/fcell.2021.639509)
Supplement: Supplementary file 1 [file Data_Sheet_1.PDF]

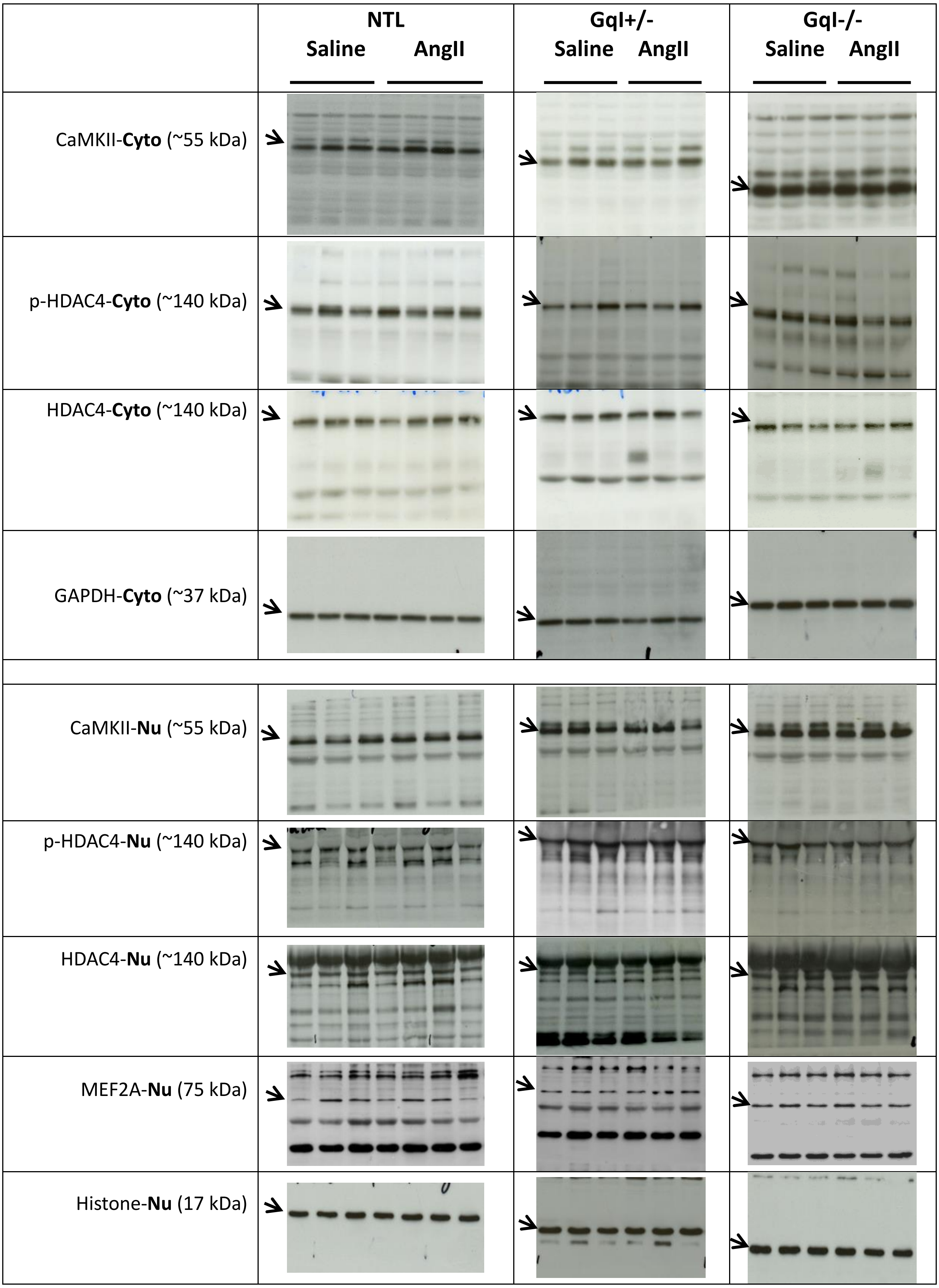

Supplementary Figure 1: Whole Western blot images for the data presented in Figure 6: CaMKII, HDAC4 and MEF2 signalling detected in the cytoplasm (upper panels) and in the nucleus (bottom panels) in response to AngII after 48 hours (n=3-4/group).
